# Development of a Method for Detection of SARS-CoV-2 Nucleocapsid Antibodies on Dried Blood Spot by DELFIA Immunoassay

**DOI:** 10.3390/diagnostics13050897

**Published:** 2023-02-27

**Authors:** Verena Damiani, Erika Pizzinato, Ilaria Cicalini, Gianmaria Demattia, Mirco Zucchelli, Luca Natale, Claudia Palmarini, Claudia Di Marzio, Luca Federici, Vincenzo De Laurenzi, Damiana Pieragostino

**Affiliations:** 1 Center for Advanced Studies and Technology (CAST), “G. d’Annunzio” University of Chieti-Pescara, 66100 Chieti, Italy; 2 Department of Innovative Technologies in Medicine and Dentistry, “G. d’Annunzio” University of Chieti-Pescara, 66100 Chieti, Italy

**Keywords:** SARS-CoV-2, nucleocapsid antibodies, ELISA, DELFIA, dried blood spot

## Abstract

Antibodies against the SARS-CoV-2 nucleocapsid protein are produced by the immune system in response to SARS-CoV-2 infection, but most available vaccines developed to fight the pandemic spread target the SARS-CoV-2 spike protein. The aim of this study was to improve the detection of antibodies against the SARS-CoV-2 nucleocapsid by providing a simple and robust method applicable to a large population. For this purpose, we developed a DELFIA immunoassay on dried blood spots (DBSs) by converting a commercially available IVD ELISA assay. A total of forty-seven paired plasma and dried blood spots were collected from vaccinated and/or previously SARS-CoV-2-infected subjects. The DBS-DELFIA resulted in a wider dynamic range and higher sensitivity for detecting antibodies against the SARS-CoV-2 nucleocapsid. Moreover, the DBS-DELFIA showed a good total intra-assay coefficient of variability of 14.6%. Finally, a strong correlation was found between SARS-CoV-2 nucleocapsid antibodies detected by the DBS-DELFIA and ELISA immunoassays (*r* = 0.9). Therefore, the association of dried blood sampling with DELFIA technology may provide an easier, minimally invasive, and accurate measurement of SARS-CoV-2 nucleocapsid antibodies in previously SARS-CoV-2-infected subjects. In conclusion, these results justify further research to develop a certified IVD DBS-DELFIA assay for detecting SARS-CoV-2 nucleocapsid antibodies useful for diagnostics as well as for serosurveillance studies.

## 1. Introduction

Today, almost three years after the severe acute respiratory syndrome coronavirus 2 (SARS-CoV-2) was first identified in China, the COVID-19 pandemic continues spreading across the world. Indeed, despite a massive vaccination campaign that has been activated against the pandemic worldwide, new highly transmissible SARS-CoV-2 variants are continuously emerging, and it is becoming increasingly clear that they may evade neutralizing antibodies generated by previous infection and/or vaccination and thus contribute to the virus circulation [1,2,3,4].

SARS-CoV-2 is an enveloped, single-stranded, positive-sense RNA virus [5]. The four main structural proteins encoded by the genome include the spike (S), membrane (M), envelope (E), and nucleocapsid (N) proteins [6]. The S protein is a trimeric protein comprising two subunits, namely S1 and S2. The S1 subunit mediates binding to host cells via interactions between its receptor-binding domain (RBD) and the human receptor angiotensin-converting enzyme 2 (ACE2), whereas the S2 subunit is responsible for membrane fusion, which is required for virus entry [7]. The N protein, in which the viral genome is encapsulated, plays a fundamental function in viral RNA transcription, replication, and virion assembly. Although the N protein is highly immunogenic and a major target for antibody response [8,9], the S protein was employed to develop vaccines first. The M and E viral structural proteins have not been investigated as vaccine targets due to their inability to induce complete immune protection; indeed, only a significant cellular immune response was elicited, whereas no robust humoral immunity was detected [10]. Currently, the majority of vaccines available on the European market target the spike protein, which is the leading immunogenic protein [11,12]. However, recently, the N protein has attracted much attention for vaccine development because it is more conservative, more stable, and has fewer mutations than the S protein [13,14,15].

In response to SARS-CoV-2 infection or vaccination, most individuals develop antibodies to the N and S proteins within 1 or 2 weeks, and these antibodies can be measured as an indicator of COVID-19 prevalence; moreover, they allow for the monitoring of seroconversion in the population and are essential elements in developing strategies for SARS-CoV-2 infection prevention and control [16,17,18]. Plasma and sera isolated from venous blood represent the conventional sample types used for the evaluation of SARS-CoV-2 antibody responses. However, the collection of these samples is invasive and requires trained personnel and equipment for immediate processing. Once collected, plasma and sera must be stored and shipped refrigerated. Therefore, dried blood spot (DBS) testing, already applied in the fields of anti-doping, toxicology, newborn screening, and the diagnosis of infectious diseases, has been validated for the measurement of SARS-CoV-2 IgG antibodies against the N and S proteins [19,20,21,22,23,24].

The most commonly used method for serological tests is the enzyme-linked immunosorbent assay (ELISA). During the SARS-CoV-2 pandemic, several ELISA methods were developed to determine SARS-CoV-2 antigens and antibodies, qualitatively and quantitatively, with great sensitivity and specificity [25,26,27]. However, a colorimetric ELISA is affected by a narrow linear range for the optical density (OD), which is common to absorbance-based measurements. For this reason, an unknown sample concentration could fall outside the standard curve, introducing the challenge of testing multiple dilutions from the same, potentially limited, sample.

The aim of this study was to develop a time-resolved fluorometry-based dissociation-enhanced lanthanide fluorescence immunoassay for detecting nucleocapsid antibodies to SARS-CoV-2 by using dried blood spots (DBS-DELFIA). The newly developed assay was compared to a commercially available, certified in vitro diagnostic (IVD) qualitative ELISA. The DBS-DELFIA test resulted in higher sensitivity and a wider dynamic range compared to the ELISA test. These results justify further research to develop a certified IVD for SARS-CoV-2 IgG anti-N detection by DBS-DELFIA technology.

## 2. Materials and Methods

### 2.1. Study Subjects

Forty-seven subjects were enrolled in this study. Sex, age, vaccination doses, and SARS-CoV-2 infection history are reported in Table S1. The study was conducted at the Center for Studies and Advanced Technologies (CAST), “G. D’Annunzio” of Chieti-Pescara, Italy, in accordance with the Declaration of Helsinki and the approval no. 16 of 1 July 2021 of the Ethics Committee of “G. D’Annunzio” University of Chieti-Pescara. Written informed consent forms were obtained from all the enrolled subjects.

### 2.2. Plasma and Dried Blood Spots Collection

Whole blood was collected via venipuncture in Vacumed sodium citrate tubes (3.2%, FL MEDICAL s.r.l., Padova, Italy) to prevent coagulation, and processed within 6 h of collection. DBS samples were prepared from venous whole blood by transferring approximately 40 µL of blood to each circle of a filter paper card. Cards were then air-dried for at least two hours, placed into bags with a desiccant dehumidifier, and stored at −20 °C. The remaining whole blood was centrifuged at 3000 rpm for 12 min. Plasma aliquots were taken and transferred into sterile microtubes and stored at −80 °C until analysis.

### 2.3. SARS-CoV-2 Nucleocapsid IgG ELISA

SARS-CoV-2 NP IgG ELISA kit [CE-IVD] (ImmunoDiagnostics, Hong Kong, China) was used following manufacturer’s recommendations. Briefly, 50 μL of negative control, 100 μL of the test sample (diluted plasma 1:100), and 100 μL of Assay Buffer (ImmunoDiagnostics) as blank were added onto the antigen-coated plate in duplicate, as the test recommended. Subsequently, the plate was incubated at room temperature (RT) for 1 h. Then, each well was manually washed 3 times with Wash Buffer (ImmunoDiagnostics) included in the kit. Next, 100 μL of Detection Solution (ImmunoDiagnostics) was added to each well and incubated for 1 h at RT. Then, after the wash step, 100 μL of Substrate Solution (ImmunoDiagnostics) was added to each well and incubated for 15 min at RT, protected from light. Finally, we added 100 μL of Stop Solution (ImmunoDiagnostics) to each well, and, after 10 min, absorbance was measured at 450 nm by Victor Nivo microplate reader (PerkinElmer, Turku, Finland).

### 2.4. Conversion from SARS-CoV-2 Nucleocapsid IgG ELISA to SARS-CoV-2 Nucleocapsid IgG DBS-DELFIA

The antigen-coated plate from SARS-CoV-2 NP IgG ELISA kit (ImmunoDiagnostics) was used. DBS disks were punched out into 3.2 mm disks by using the PerkinElmer DBS Puncher, while plasma samples were diluted 1:100 with DELFIA Assay Buffer (PerkinElmer). Next, DBS disks were extracted with 100 μL DELFIA Assay Buffer directly onto the antigen-coated plate, whereas 100 μL of diluted plasma was transferred to the plate, and both DBS disks and plasma samples were incubated for 2 h at RT on a plate shaker set at 300 rpm. Then, after removing DBS disks and plasma samples, each well was manually washed 4 times with DELFIA Wash Solution (PerkinElmer). Next, 100 μL (200 ng/mL) of DELFIA Eu-labeled Anti-human IgG antibody (PerkinElmer) was added to each well and incubated for 1 h at RT on a plate shaker set at 300 rpm. Subsequently, each well was washed 6 times with DELFIA Wash Solution. Finally, 200 μL of DELFIA Enhancement Solution (PerkinElmer) was added, and the plate was read after 10 min of incubation time by Victor Nivo microplate reader using fluorescence (TRF) settings.

### 2.5. Linearity, Precision Study, and Statistical Analysis

To test the linearity of both ELISA and DBS-DELFIA immunoassays, a SARS-CoV-2 anti-N IgG positive sample was diluted sequentially 12 times with a seronegative plasma and tested in duplicate. Dilution percentages are listed in Table S2. Data for linearity and intra-assay precision were collected by the same operator. All statistical analyses were carried out using GraphPad Prism 9.0.2 software (GraphPad Software, La Jolla, CA, USA).

## 3. Results

### 3.1. Conversion from ELISA to DBS-DELFIA Immunoassay for SARS-CoV-2 Nucleocapsid Antibody Detection

The study population included fifteen subjects who had never tested positive for SARS-CoV-2 infection, thirty-two subjects who reported a positive nasopharyngeal swab (NPS) test, and forty-three individuals who had completed the vaccination schedule. The age of the subjects ranged from 25 to 60 (mean = 35.6), and 70% were female (Table S1). Firstly, we conducted an experiment to set up the method for converting ELISA into DBS-DELFIA by using paired plasma samples and dried blood spots from two subjects, one negative and one positive for SARS-CoV-2 infection. The cut-off value used by the qualitative SARS-CoV-2 nucleocapsid antibody ELISA kit was 0.2 OD. We analyzed, in parallel and in duplicate, the plasma diluted 100 times both by ELISA and by DELFIA, and the paired DBSs by DELFIA following the manufactures’ instructions (Supplementary Table S3). The positive subject, with 2.47 ± 0.06 OD, had almost comparable values for plasma DELFIA and DBS-DELFIA (510,238 ± 11,641.8 TRF and 496,748 ± 96,228.75 TRF, respectively). On the other hand, the negative subject showed different values between plasma DELFIA and DBS-DELFIA (24,067.5 ± 678.11 TRF and 93,547.5 ± 12,087.99 TRF). For this reason, we attempted to improve the DBS-DELFIA protocol using the commercially available ELISA-to-DELFIA conversion kit protocol distributed by PerkinElmer, which is fully described in the Section 2.

### 3.2. DBS-DELFIA Method Linearity and Performance Assessment

**Figure 1:**
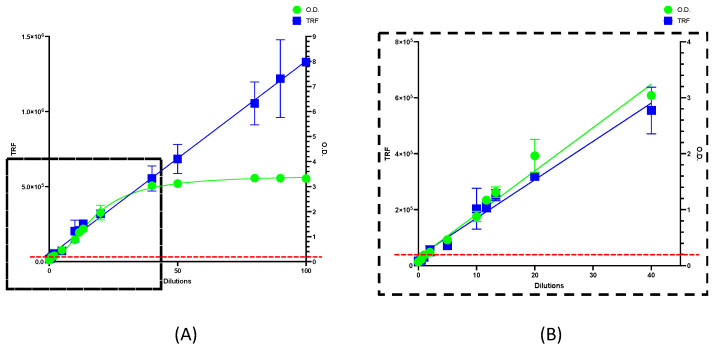
DBS-DELFIA vs. ELISA dilutions linearity range. (**A**) DBS-DELFIA dilutions results are expressed as TRF, whereas ELISA results are expressed as OD values. (**B**) Magnification of rectangular black dashed section highlighting ELISA linearity range. Red dashed line expressed ELISA cut-off value (0.2 OD).

To prove the linearity of the method for quantifying IgG anti-N, we performed sequential dilutions using two blood samples, one that tested negative (dilution percentage 0, 0.07 OD) and one that tested positive (dilution percentage 100, 3.33 OD) for anti-SARS-CoV-2 nucleocapsid antibodies by the ELISA method. We analyzed 12 paired dilutions by both ELISA and the new DBS-DELFIA method, using different sample matrices (plasma or DBS) (Table S2). The DELFIA method had a wider dynamic range than conventional ELISA (Figure 1). While the linear range of DELFIA covered dilution percentages up to 100 with an R2 equal to 0.97, the ELISA method only showed linearity below the 40-dilution percentage (R^2^ = 0.97).

Next, we examined the cut-off value referred to in the ELISA kit and noted that for 1:100 (0.191 ± 0.03 OD) and 2:100 (0.241 ± 0.03 OD) dilutions, the test resulted in negative and positive results, respectively. However, the paired samples analyzed by DBS-DELFIA measured 30,351 ± 4912 TRF and 53,652 ± 17,674 TRF, showing a higher sensitivity potential. In order to evaluate intra-assay precision, we evaluated the coefficient of variability in percentage (CV%) of both obtained curves. We obtained 7% and 15.2 CV% for ELISA and DBS-DELFIA, respectively. Finally, we calculated the Limit of Detection (LOD) and Limit of Quantification (LOQ) for both methods by using blank sample replicates (*n* = 8), obtaining 0.09 and 0.18 values, respectively, for the ELISA immunoassay, and 2797 and 3787 values for the DBS-DELFIA one.

### 3.3. Evaluation of SARS-CoV-2 IgG Anti-N Detection by ELISA vs. DBS-DELFIA Immunoassay

**Figure 2:**
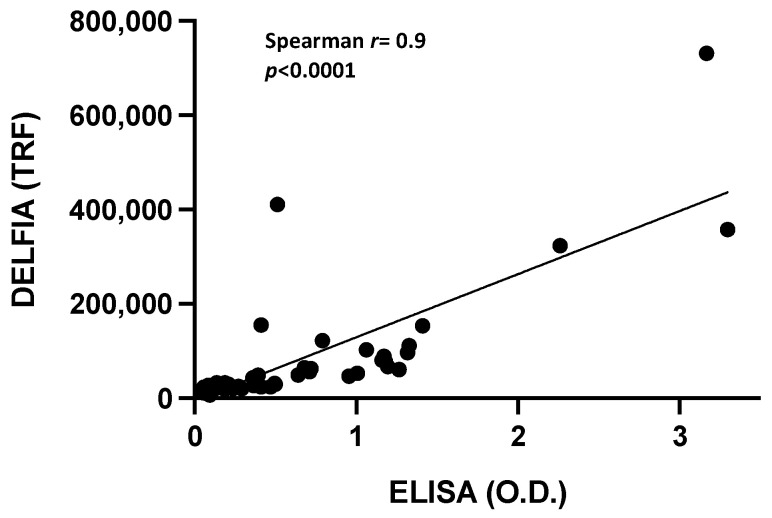
Correlation between DBS-DELFIA and plasma ELISA for SARS-CoV-2 nucleocapsid IgG antibody detection.

All paired plasma samples and dried blood spots were collected and analyzed by both ELISA and DBS-DELFIA immunoassays for the detection of SARS-CoV-2 nucleocapsid antibodies (Table S4). A significant positive correlation (*r* = 0.9, *p* < 0.0001) between IgG anti-N measured on DBS and plasma with DELFIA and ELISA immunoassays, respectively, was observed (Figure 2).

**Figure 3:**
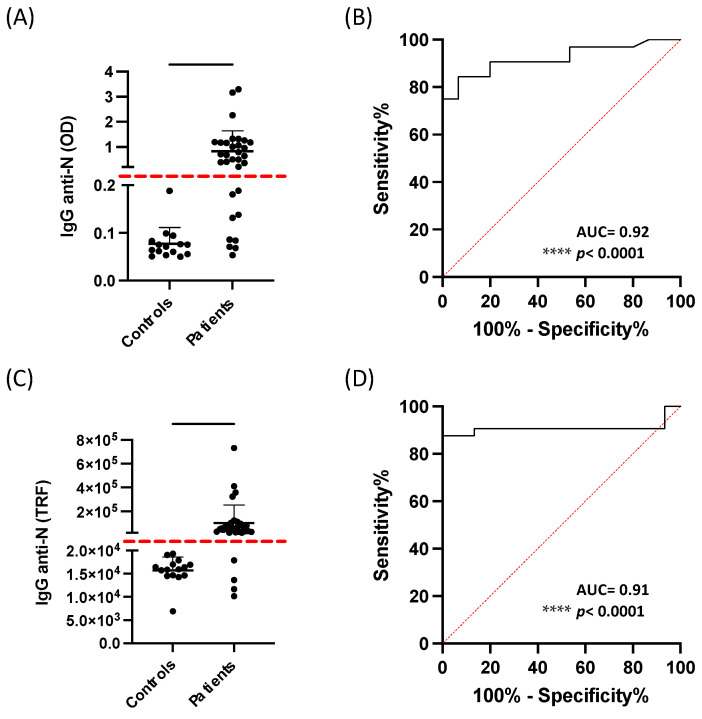
ELISA vs. DBS-DELFIA performance analysis. (**A**,**C**) Scatter plots and (**B**,**D**) receiver operating characteristic (ROC) curves for SARS-CoV-2 nucleocapsid antibody detection by ELISA and DBS-DELFIA, respectively. IgG anti-N concentration in each subject. The plots show mean ± standard deviation. ROC curves indicate the area under the curve (AUC). **** *p* < 0.0001.

Fifteen subjects had never tested positive for SARS-CoV-2 infection using NPS. At the same time, thirty-two subjects tested positive at different times after being tested for SARS-CoV-2 nucleocapsid antibodies. All subjects without SARS-CoV-2 infection tested negative on the ELISA test (OD < 0.2). Overall, these subjects presented a mean TRF below 2.0 × 10^4^. Despite significant differences in IgG anti-N levels evaluated by both ELISA and DBS-DELFIA between subjects who were negative or positive for SARS-CoV-2 infection (Figure 3A,C), the receiver operator characteristic (ROC) curve analysis revealed higher sensitivity (87.5%, AUC: 0.91, *p* < 0.0001) for the DBS-DELFIA assay compared to ELISA (71.88%, AUC: 0.92, *p* < 0.0001) while the specificity was 100% for both assays (Figure 3B,D). Moreover, the ROC analysis showed that 87.5% sensitivity was achieved above 20,120 TRF; therefore, we set the cut-off value for the DBS-DELFIA assay at 2.0 × 10^4^ (Figure 3C). Five samples (PZ 24, 25, 32, 44, and 47) tested negative when analyzed with ELISA but appeared above the assessed cut-off value when analyzed with the DBS-DELFIA method. By calculating the percentage difference between the cut-off value and LOQ for both evaluated methods, we observed that the ELISA test’s positivity limit was 2% higher than the LOQ, while the DBS-DELFIA positivity limit was more than 80% higher than its relative LOQ.

## 4. Discussion

With thousands of new cases daily, the ongoing scenario indicates that the SARS-CoV-2 pandemic will continue to evolve [28]. Indeed, as SARS-CoV-2 continues to spread in human populations with fewer susceptible hosts, the risk of selecting more infectious variants or antibody-evasive mutations is expected to increase. Even to avoid undiagnosed cases of SARS-CoV-2 infection in emergencies [29], viral tests are used for the assessment of current infection with SARS-CoV-2 by testing respiratory tract specimens (throat swabs, sputum, nasopharyngeal swabs, nasal swabs, and bronchoalveolar lavage fluid). There are two main types of viral tests: nucleic acid amplification tests (NAATs, such as reverse transcription polymerase chain reaction) and antigen tests [30]. However, the collection of samples from the respiratory tract is relatively complicated and causes significant discomfort to subjects. Moreover, the costs of NAATs continue to remain high. Therefore, growing interest has been placed in developing serological tests for the detection of anti-SARS-CoV-2 antibodies to help identify people who have been infected with SARS-CoV-2, have recovered from COVID-19, or have been vaccinated [31,32]. Notably, DBS specimens have been seen to be reliably used as an alternative to serum samples for SARS-CoV-2 antibody measurement, facilitating serosurveillance efforts [33,34]. We have already validated the GSP^®®^/DELFIA^®®^ Anti-SARS-CoV-2 IgG Kit for measuring anti-S1 antibodies by using DBS, demonstrating the feasibility of such serological tests for high-throughput serosurveillance [35]. However, to ascertain whether a recent SARS-CoV-2 infection occurred among subjects who have previously been vaccinated for the prevention of COVID-19, high-sensitivity immunoassays for SARS-CoV-2 anti-nucleocapsid antibodies are required. Notably, there is evidence of longer durability of anti-spike antibodies after vaccination with the mRNA vaccine in subjects with previous infection, and the risk of new SARS-CoV-2 infection appears to be higher in previously uninfected individuals [36,37]. This is why knowing the SARS-CoV-2 nucleocapsid antibody profile is extremely important.

For this purpose, we established a dissociation-enhanced lanthanide fluorescence (DELFIA) immunoassay for the evaluation of SARS-CoV-2 anti-nucleocapsid IgG antibody status by analyzing dried blood spots (DBS-DELFIA). We converted a commercially available CE-IVD SARS-CoV-2 nucleocapsid protein IgG ELISA kit validated with serum or plasma samples into the DBS-DELFIA. Our results confirm a wider dynamic range and higher sensitivity of the DBS-DELFIA compared to the ELISA immunoassay in recognizing IgG anti-N against SARS-CoV-2, revealing lower amounts of antibodies even when several days have passed since the previous infection. Moreover, the total intra-assay coefficient of variability of the DBS-DELFIA was 14.6%, indicating good assay precision. Finally, a strong correlation between SARS-CoV-2 nucleocapsid antibodies detected by the DBS-DELFIA and ELISA immunoassays was found (*r* = 0.9). Therefore, the association of dried blood sampling with DELFIA technology, in addition to a simple, non-invasive approach and little time-consuming sample preparation, may provide a more sensitive and accurate measurement of SARS-CoV-2 nucleocapsid antibodies than tests currently available, particularly for low detectable or higher quantizable antibody levels among SARS-CoV-2-infected subjects, with the further potential of being fully automated [35].

## 5. Conclusions

In summary, we developed a new serological immunoassay specifically for the detection of SARS-CoV-2 nucleocapsid antibodies employing DELFIA technology. The assay showed increased sensitivity and appears to be particularly suitable for high-throughput serosurveillance studies, thus justifying further research aimed at developing a certified IVD DBS-DELFIA assay for detecting SARS-CoV-2 nucleocapsid antibodies.

## Data Availability

The data presented in this study are available in Supplementary Materials.

## Supplementary Materials

The following supporting information can be downloaded at: https://www.mdpi.com/article/10.3390/diagnostics13050897/s1, Table S1: Clinical parameters of the 47 plasma-DBS paired samples collected. Table S2: Linearity assessment of ELISA and DBS-DELFIA methods analyzing SARS-CoV-2 IgG anti-N on sequential dilutions. Table S3: Data of the set-up experiment for ELISA to DELFIA and next to DBS-DELFIA conversion. Table S4: Data of analytical sensitivity of ELISA and DBS-DELFIA immunoassays used to evaluate assay precision.
