# Supplementary material for: Development of a Method for Detection of SARS-CoV-2 Nucleocapsid Antibodies on Dried Blood Spot by DELFIA Immunoassay
Source: Diagnostics (Basel). 2023 Feb 27;13(5):897. doi: 10.3390/diagnostics13050897 (PMC10000641; doi:10.3390/diagnostics13050897)
Supplement: Supplementary file 1 [file diagnostics-13-00897-s001.zip › diagnostics-2225626-supplementary.pdf]

Supplementary materials of

Improvement in detecting SARS-CoV-2 nucleocapsid antibodies by DELFIA immunoassay in dried blood spots

Verena Damiani [1,2,*], Erika Pizzinato [1,2], Ilaria Cicalini [1,2], Gianmaria Demattia [1], Mirco Zucchelli [1,2], Luca Natale [1], Claudia Palmarini [1], Claudia Di Marzio [1], Luca Federici [1,2], Vincenzo De Laurenzi [1,2] and Damiana Pieragostino [1,2]

[1] Center for Advanced Studies and Technology (CAST), "G. d'Annunzio" University of Chieti-Pescara, 66100 Chieti, Italy

[2] Department of Innovative Technologies in Medicine and Dentistry, "G. d'Annunzio" University of Chieti-Pescara, 66100 Chieti, Italy

* Correspondence: verena.damiani@unich.it, Tel. +390871355582

Supplementary Table S1. Clinical parameters of the 47 plasma-DBS paired samples collected.

Subjects	Gender	Age	Date of SARS-CoV-2 infection	Vaccination Doses	Date of sample colletion	Days after NPS
Pz1	M	60	-	3	30/08/2022	-
Pz2	F	28	-	3	27/05/2022	-
Pz3	F	28	-	3	27/05/2022	-
Pz4	F	28	-	3	27/05/2022	-
Pz5	F	30	-	3	27/05/2022	-
Pz6	F	33	-	3	27/05/2022	-
Pz7	M	40	-	3	27/05/2022	-
Pz8	F	45	-	3	27/05/2022	-
Pz9	F	45	-	3	27/05/2022	-
Pz10	M	29	-	3	29/04/2022	-
Pz11	F	26	-	3	29/04/2022	-
Pz12	F	28	-	3	29/04/2022	-
Pz13	F	40	-	3	18/10/2022	-
Pz14	M	50	-	3	29/04/2022	-
Pz15	F	35	-	3	20/09/2022	-
Pz16	F	40	21/04/2022	3	27/05/2022	36
Pz17	F	28	29/01/2022	2	27/05/2022	119
Pz18	F	30	02/05/2022	3	27/05/2022	22
Pz19	F	30	09/04/2022	3	27/05/2022	48
Pz20	F	37	17/04/2022	3	27/05/2022	40
Pz21	F	27	08/04/2022	3	27/05/2022	49
Pz22	F	26	16/12/2021	2	27/05/2022	162
Pz23	M	45	22/04/2022	3	27/05/2022	35
Pz24	F	40	05/04/2022	3	27/05/2022	52
Pz25	M	50	22/02/2022	3	27/05/2022	94
Pz26	F	47	11/04/2022	3	27/05/2022	46
Pz27	F	42	24/04/2022	3	27/05/2022	33
Pz28	M	30	14/04/2022	3	27/05/2022	43
Pz29	F	35	05/03/2022	3	29/04/2022	55
Pz30	M	30	14/04/2022	3	29/04/2022	15
Pz31	M	40	10/02/2022	3	29/04/2022	78
Pz32	F	40	05/04/2022	3	29/04/2022	24
Pz33	F	40	30/03/2022	3	29/04/2022	30
Pz34	F	35	12/04/2022	3	29/04/2022	17
Pz35	M	25	28/03/2022	3	29/04/2022	32
Pz36	F	26	03/03/2021	2	29/04/2022	422
Pz37	F	40	15/04/2022	3	29/04/2022	14
Pz38	M	28	08/04/2022	3	29/04/2022	21
Pz39	F	28	19/04/2022	3	29/04/2022	10
Pz40	F	30	06/01/2022	3	29/04/2022	113
Pz41	F	45	29/04/2021	3	29/04/2022	365
Pz42	M	26	03/03/2021	2	29/04/2022	422
Pz43	M	30	28/12/2021	3	29/04/2022	122
Pz44	F	28	10/02/2022	3	27/09/2022	230
Pz45	F	40	21/04/2022	3	28/09/2022	160
Pz46	M	50	15/07/2022	3	28/09/2022	75
Pz47	F	40	05/04/2022	3	28/09/2022	177

NPS: nasopharyngeal swab.

Supplementary Table S2. Linearity assessment of ELISA and DBS-DELFIA methods analyzing SARS-CoV-2 IgG anti-N on sequential dilutions.

Dilution percentage	ELISA (OD)	DBS-DELFIA (TRF)
100	3.3255 ± 0.002	1143229 ± 264386.5
90	3.3345 ± 0.002	1219575 ± 258587.5
80	3.3395 ± 0.009	1054604.5± 143594.3
50	3.1235 ± 0.027	685142.5 ± 96684.83
40	3.042 ± 0.019	554589.5 ± 83251.22
20	1.9635 ± 0.293	318760 ± 7471.29
13.3	1.3125 ± 0.094	252103.5 ± 20023.14
11.7	1.1705 ± 0.058	208391.5 ± 16283.96
10	0.88 ± 0.091	203548 ± 73194.04
5	0.462 ± 0.046	71840.5 ± 6482.05
2	0.241 ± 0.033	58652 ± 10674.84
1	0.191 ± 0.026	30351 ± 4912.97
0.5	0.114 ± 0.007	18839 ± 1179.45
0	0.0695 ± 0.026	179098 ± 214.96

Values are presented as mean ± SD.

Supplementary Table S3. Data of the set-up experiment for ELISA to DELFIA and next to DBS-DELFIA conversion.

	ELISA (OD)	DELFIA(plasma)	DBS-DELFIA
NEG	0.144 ± 0.02	24067.5 ±678.11	93547.5 ± 12087.99
POS	2.47 ± 0.06	510238 ± 11641.8	496748 ± 96228.75

Values are presented as mean ± SD. POS: subject tested positive for SARS-CoV-2 IgG anti-N. NEG: subject tested negative for SARS-CoV-2 IgG anti-N.

Supplementary Table S4. Data of analytical sensitivity of ELISA and DBS-DELFIA immunoassays used to evaluate assay precision.

Subjects	OD	ELISA CV%	TRF	DBS-DELFIA CV%
Pz1	0,099 ± 0,02	19,99897967	14603,5 ± 2034,85	13,93053863
Pz2	0,05 ± 0,01	11,3137085	15729,5 ± 2322,85	14,76744827
Pz3	0,051 ± 0,00	5,545935539	14264 ± 479,4	3,361037561
Pz4	0,0555 ± 0,00	3,822198817	16418 ± 1678,7	10,22457972
Pz5	0,0605 ± 0,00	3,506314617	15875,5 ± 557,9	3,514265695
Pz6	0,0535 ± 0,00	1,321694918	14637 ± 1377,44	9,41069898
Pz7	0,0755 ± 0,01	8,429087458	16915,5 ± 2334,2	13,79893875
Pz8	0,0755 ± 0,00	4,682826365	16996,5 ± 1668,1	9,814167016
Pz9	0,062 ± 0,01	9,123958467	17855 ± 569,9	3,191980205
Pz10	0,064 ± 0,01	8,838834765	14500,5 ± 4673,3	32,2283281
Pz11	0,0945 ± 0,00	3,741305721	6911,5 ± 589,02	8,522317134
Pz12	0,0765 ± 0,00	0,92432259	16384 ± 1977,1	12,06708106
Pz13	0,083 ± 0,01	15,33484586	19029,5 ± 5130,1	26,95845764
Pz14	0,188 ± 0,01	3,761206283	19272,5 ± 381,1	1,977587521
Pz15	0,0715 ±0,00	6,922723732	15924 ± 1146,9	7,202506902
Pz16	**1,3275 ± 0,06**	4,847210327	**111569 ± 19076,33**	17,09823225
Pz17	**1,1945 ± 0,11**	8,938729507	**66692 ± 6301,74**	9,449012826
Pz18	**1,264 ± 0,11**	8,950718749	**61184 ± 17591,4**	28,7516385
Pz19	**1,179 ± 0,02**	1,559353377	**78162,5 ± 14902,28**	19,06576096
Pz20	**1,0055 ± 0,03**	2,883279764	**53203,5 ± 19945,36**	37,48881366
Pz21	**0,4115 ± 0,01**	1,546527589	**24065 ± 534,57**	2,22137015
Pz22	**2,26 ± 0,03**	1,251516427	**323392,5 ± 108295,5**	33,48733346
Pz23	**0,6405 ± 0,03**	4,747164963	**49478,5 ± 902,98**	1,824985316
Pz24	0,181 ± 0,01	3,906667299	**20967 ± 1525,95**	7,277800514
Pz25	0,1885 ± 0,02	8,627828099	**32906 ± 1552,8**	4,718915977
Pz26	**1,1605 ± 0,02**	1,401418007	**80897,5 ± 4813,3**	5,949845001
Pz27	0,086 ± 0,01	11,51104062	17860 ± 1452,4	8,1321239
Pz28	**1,06315 ± 0,07**	6,671006928	**102720 ± 32567,9**	31,70553361
Pz29	**0,79 ± 0,04**	4,65437375	**122513 ± 30289,6**	24,72360164
Pz30	**0,394 ± 0,01**	2,871499619	**49161 ± 11780,4**	23,96289533
Pz31	**0,209 ± 0,00**	2,029971621	**29539 ± 9090,6**	30,77478851
Pz32	0,084 ± 0,00	0	**27360 ± 507,7**	1,85563841
Pz33	**0,6795 ± 0,02**	2,393444587	**64737 ± 13457,7**	20,7881988
Pz34	**0,499 ± 0,00**	0	**29567,5 ± 6129,9**	20,73191405
Pz35	**0,711 ± 0,05**	6,762765277	**56184,5 ± 13461,2**	23,95890645
Pz36	0,071 ± 0,00	3,983700176	10154,5 ± 2331,33	22,9586002
Pz37	**0,3605 ± 0,04**	12,35720588	**43811 ± 15793,94**	36,05016335
Pz38	**1,318 ± 0,12**	9,442397078	**96709 ± 626,5**	0,647816241
Pz39	0,0685 ± 0,00	3,09681802	13628,5 ± 801,15	5,87850448
Pz40	**3,167 ± 0,02**	0,580510777	**731559,5 ± 208206,88**	28,46069043
Pz41	0,0535 ± 0,00	1,321694918	11663 ± 428,51	3,67406936
Pz42	**0,512 ± 0,03**	5,800485314	**410844 ± 10726,81**	2,610920415
Pz43	**1,172 ± 0,05**	4,223334017	**88704,5 ± 12679,13**	14,29367359
Pz44	0,1315 ± 0,01	11,29219955	**21160,5 ± 5699,99**	26,93692381
Pz45	**0,9565 ± 0,02**	2,29172088	**47251,5 ± 2976,21**	6,298662354
Pz46	**3,2965 ± 0,01**	0,235952513	**357352,5 ± 31172,8**	8,72326413
Pz47	0,138 ± 0,03	19,47105629	**32807 ± 784,89**	2,392442244

Values are presented as mean ± SD.
